# Rho-Kinase as a Target for Cancer Therapy and Its Immunotherapeutic Potential

**DOI:** 10.3390/ijms222312916

**Published:** 2021-11-29

**Authors:** Seohyun Kim, Seong A. Kim, Jihoon Han, In-San Kim

**Affiliations:** 1 KU-KIST Graduate School of Converging Science and Technology, Korea University, Seoul 02841, Korea; kkksh03@shiftbio.net (S.K.); 091989@kist.re.kr (S.A.K.); jihoonhan@kist.re.kr (J.H.); 2 Center for Theragnosis, Biomedical Research Institute, Korea Institute of Science and Technology (KIST), Seoul 02792, Korea

**Keywords:** Rho-kinase (ROCK), cancer immunotherapy, ROCK inhibitors, tumor microenvironment (TME)

## Abstract

Cancer immunotherapy is fast rising as a prominent new pillar of cancer treatment, harnessing the immune system to fight against numerous types of cancer. Rho-kinase (ROCK) pathway is involved in diverse cellular activities, and is therefore the target of interest in various diseases at the cellular level including cancer. Indeed, ROCK is well-known for its involvement in the tumor cell and tumor microenvironment, especially in its ability to enhance tumor cell progression, migration, metastasis, and extracellular matrix remodeling. Importantly, ROCK is also considered to be a novel and effective modulator of immune cells, although further studies are needed. In this review article, we describe the various activities of ROCK and its potential to be utilized in cancer treatment, particularly in cancer immunotherapy, by shining a light on its activities in the immune system.

## 1. Introduction

The concept of cancer immunotherapy was first suggested with the plan to harness the patient immune system toward malignant tumor antigens in order to combat cancer. This strategy is fast becoming a revolutionary paradigm in cancer treatment, overcoming immune obstacles such as evasion of immune detection through the increase in immune checkpoints, and other mechanisms of immunosuppression tumor cells may develop. In the past few years, cancer immunotherapy has made leaps and bounds due to a better understanding of immune surveillance and usage of immune checkpoints including cytotoxic T-lymphocyte-associated protein 4 (CTLA-4) and programmed cell death protein 1 (PD-1). Discoveries of antibodies blocking such immune checkpoints have resulted in a dramatic clinical improvement in patients with various cancer types such as melanoma [1], Hodgkin lymphoma [2], and Merkel cell carcinoma [3]. Moreover, cancer immunotherapy not only offers a high response rate, but also long-term cancer remission through a persisted antitumor immunity that can induce antitumor memory against a possible relapse. Due to the numerous advantages and efficacy of immunotherapy, it is fast becoming a first line indication for the treatment of cancer. However, immunotherapies based on immune checkpoint blockade only work in tumors with a T cell-abundant microenvironment, and poorly immunogenic tumors lacking T cell infiltration usually fail to respond. Therefore, discovery of other therapeutic strategies is necessary in order for immune-excluded tumors as well as immune desert tumors to be responsive [4,5]. Kinase signaling pathways are known to be involved in the regulation of tumor immunity. Targeting kinases in cancer cells and immune cells, alone or in combination with other therapies, has emerged to be a promising immunotherapeutic strategy in oncology [6].

Rho-kinase (ROCK) is a downstream effector of the small guanosine triphosphatase (GTPases), RhoA, B, and C. ROCK is involved in multiple cellular activities by regulating the actin cytoskeleton including cell contraction, motility, morphology, and proliferation. Therefore, ROCK is suggested to be a therapeutic target in numerous diseases such as cardiovascular diseases, ophthalmic diseases [7], neurological disorders [8], autoimmune diseases [9], and cancer [10]. Discovery and development of molecular inhibitors that selectively target ROCK1, ROCK2, or both, carry high clinical value [11]. Recently, two novel pharmacologic compounds, belumosudil (Rezurock, 2021) and netarsudil (Rocklatan, 2019) that inhibit ROCK with high selectivity and activity, have been approved for clinical use. Belumosudil was approved for the treatment of chronic graft-versus host disease while netarsudil, in combination with prostaglandin F_2α_ analogue latanoprost, was approved for the reduction in elevated intraocular pressure (IOP) in patients with glaucoma or ocular hypertension. Ripasudil [12] and fasudil [13], both ROCK inhibitors, were each clinically approved in Japan for glaucoma and cerebral vasospasm, respectively. Additionally, for cancer treatment, a phase 1 clinical trial using AT13148, the first dual potent ROCK-AKT kinase (AKT) inhibitor, was completed in 2018 for the treatment of advanced solid tumors. However the administration route and combinational treatment agents should be further considered, in its side effects and limited clinical efficacy (NCT01585701) [14] Clinical trials on ROCK inhibitors have been under way for various indications as described in Table 1.

The role of ROCK in various cellular activities naturally indicates its involvement in multiple steps of tumor progression. ROCK simultaneously controls complex tumor environmental components including stromal cells and immune cells. Numerous studies have explored the potential of ROCK-targeted therapy for cancer treatment due to its multiple functions in various cell types in the tumor microenvironment (TME). Considering this, ROCK inhibitors could be a potent therapeutic option. However, beyond the therapeutic function of ROCK in cancer cells, its influence on immune cells and the corresponding anti-tumor immune responses remain to be elucidated yet. Therefore, with emerging interests in cancer immunotherapeutic regimens, understanding the role of ROCK in the TME is essential for applying ROCK inhibitors in cancer immunotherapy. In this review, we focus on the biological functions of ROCK in various cell types, demonstrating its therapeutic usages as a cancer treatment modality, and suggesting its immunotherapeutic potential to modulate the immunopathology in the TME.

## 2. Rho-Kinase (ROCK)

Rho-kinase (ROCK) was originally discovered to specifically bind to GTP-bound RhoA and act as a serine/threonine kinase [14,15]. Belonging to the AGC protein kinase family, ROCK consists of a kinase domain at the N-terminus, followed by a coiled-coil region and a pleckinstrin homology domain at the C-terminus [16]. This COOH terminus of ROCK acts as an auto-inhibitory region [17]. ROCK has two isoforms, ROCK1 and ROCK2, with distinct expression patterns, where mRNA of ROCK1 is ubiquitously expressed in most organs except for brain and muscle tissues, while that of ROCK2 is expressed only in certain organs such as the brain, heart, muscle, and lung [18]. ROCK can be activated in a Rho-dependent or independent manner. In a Rho-dependent ROCK activation, activated Rho GTPase family proteins including RhoA, RhoB, and RhoC interact with ROCK with different affinities, most likely due to minor differences in their sequences [19]. In a Rho-independent manner, ROCK1 and ROCK2 can be activated by cleavage of the auto-inhibitory C-terminus, mediated by caspase3 and caspase2, respectively [20,21]. ROCK2 can also be directly activated by granzyme B [22]. Negative regulations of ROCK1 by Gem GTPase and RhoE and ROCK2 by Coronin1B have also been well characterized [23].

**Figure 1:**
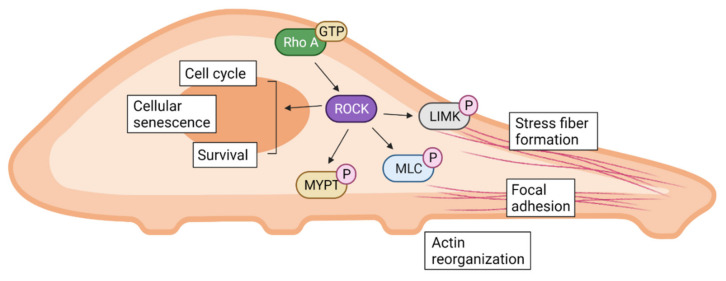
The Rho/ROCK signaling pathway in cell biology. The Rho/ROCK signaling regulates fundamental cellular activities including actin reorganization, focal adhesion, and stress fiber formation, altering various cellular states.

ROCK has been shown to regulate various biological functions as illustrated graphically in Figure 1. Conventionally, the Rho/ROCK signaling pathway is generally recognized as an important regulator for cell cytoskeleton and polarity [24]. ROCK directly phosphorylates myosin light chain (MLC) and inactivates myosin phosphatase, regulating cell contraction in smooth muscle cells as well as non-muscle cells [25]. ROCK also interacts with specific substrates involved in reorganization of the cell cytoskeleton such as LIM kinase (LIMK), adducin, and vimentin, which can lead to actin reorganization, focal adhesion, and stress fiber formation [26,27]. ROCK1 and ROCK2 both show the ability to distinctly regulate stress fiber disassembly and cell detachment as well as adhesion complex assembly and keratinocyte turnover [28,29]. They also individually contribute to the assembly of the fibronectin matrix and myosin II-driven contractility, but not in stress fibers [30].

Along with its ability to regulate the cell cytoskeleton, the Rho/ROCK signaling pathway is also involved in cell migration [31]. In endothelial cells, hyaluronan-stimulated ROCK is activated and consequently increases the phosphorylation of phosphatidyl inositol receptors, stimulating a Ca^2+^ influx to induce cell migration [32]. In human neutrophils, ROCK1 was shown to regulate their migration via phosphorylation of MLC in vitro [33]. Under the stimuli of platelet-derived growth factor and lysophosphatidic acid, the Rho/ROCK pathway regulated the cell migration of smooth muscle cells either with or without the involvement of MLC phosphorylation [34]. The Rho/ROCK signaling pathway is also closely related to cell death and survival. For instance, the morphological changes in apoptotic cells heavily requires the interference of ROCK [35]. Furthermore, along with cytoskeletal regulation, ROCK is also related to cell cycle progression, involving mitosis, chromosome alignment, and segregation of daughter cells [36]. On a similar note, both ROCK1 and ROCK2 regulate cell proliferation, and inhibiting them leads to cell cycle arrest and cellular senescence [37].

## 3. Targeting ROCK for Cancer Treatment

The role of ROCK in cancer has been widely explored, especially in fields of cancer development, progression, and metastasis [38,39]. For example, in advanced stages of breast cancer, ROCK has usually been shown to be overexpressed or of higher activity [40]. High expression of ROCK1 was observed in the metastasis of breast cancer, increasing the invasiveness of cancer cells. ROCK1 overexpression is also negatively correlated with the survival rate of breast cancer patients [41]. Likewise, an amplification of *ROCK1* and *ROCK2* expression was observed in pancreatic cancer [42]. In colorectal cancer (CRC), *ROCK* polymorphism was shown to correlate with cancer development, again indicating the contribution of ROCK in cancer growth [43]. Overexpression of *ROCK2* was also observed in hepatocellular carcinoma, intimately associated with tumor microsatellite formation, further enabling tumor migration and invasion [44].

High expression of ROCK is associated with poor prognosis in medulloblastoma [45] neuroblastoma [46], and vascular tumors [47]. In medulloblastoma including metastatic medulloblastoma, mRNA levels of *ROCK*, especially *ROCK2*, were highly expressed, which was associated with the overall survival of patients. ROCK inhibition with RKI-1447 showed less tumorigenicity through the inhibition of ROCK-dependent cell migration and invasion. Gene expressions illustrated that ROCK inhibition downregulates tumor necrosis factor α (TNFα) via nuclear factor kappa-light-chain-enhancer of activated B cells (NF-κB) and epithelial–mesenchymal transition (EMT) signaling associated with medulloblastoma progression and metastasis [45]. Neuroblastoma patients were found to harbor somatic mutations in Rho/Rac signaling and high expression levels of ROCK2, which was associated with poor patient survival. Inhibition of ROCK2 was shown to significantly suppress tumor invasiveness, differentiation, and growth of neuroblastoma, either with or without amplification of the *MYCN* gene, which is a strong oncogenic driver inducing neoplastic transformation. For example, ROCK2 inhibition using HA1077 induced phosphorylation of Thr58 of MYCN by the dephosphorylation of Ser9 of glycogen synthase kinase 3β (GSK3β), leading to proteosomal degradation of MYCN [46]. Vascular tumors including hemangiomas and angiosarcomas, also harbor high expression of ROCK relative to normal cells. Knockdown of *ROCK2*, but not *ROCK1*, exhibited tumor growth inhibition in xenograft animal models due to the contribution of ROCK2 to tumor cell survival [47]. However, the role of ROCK and the effects of its genetic ablation in cancer development and progression still needs to be fully uncovered.

**Figure 2:**
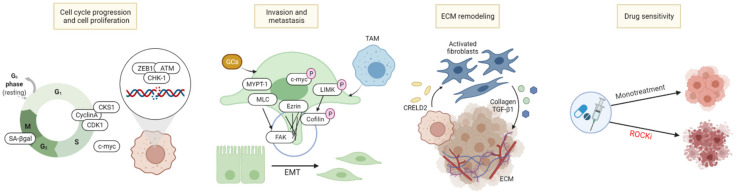
Cancer treatment strategies involving ROCK. ROCK is involved in multiple steps of the cell cycle and DNA damage response. ROCK further plays an important role in all stages of cancer cell metastasis including the formation of filopodia and lamellipodia, cytoskeletal reprogramming, EMT process, and the regulation of TME components. ROCK also activates pro-tumorigenic CAFs and increases the deposition of ECM components. This increase in ECM deposition promotes its stiffness and rigidity to exert tumor cell progression. Finally, ROCK inhibition combined with other chemotherapeutic agents can induce a synergistic antitumor effect.

As ROCK and its upstream and downstream signaling molecules control the motility, migration, and survival in various cells, ROCK is involved in the progression and invasiveness of cancer cells, and is closely related to the components of the TME including tumor cells, immune cells, and extracellular matrix (ECM). Based on its various activities, there are several strategies to involve ROCK in cancer treatment, as illustrated in Figure 2.

### 3.1. Cell Cycle Arrest/Cell Death Induction

The role of Rho-kinase in cell proliferation and death is well demonstrated, and numerous studies have investigated the anti-tumor effects of ROCK inhibition in vitro and in vivo. ROCK regulates actin polymerization and cytoskeletal dynamics, thus, inhibition of ROCK1 and ROCK2 reduces actomyosin contractility, and induces phenotypical change of cells into a flattened morphology. Inhibition of Rho-kinase causes a variety of changes in cell morphology, shifting it from regular to irregular such as lamellipodia formation decrease, cell body elongation, and conversion to a spindle-like cellular shape [48,49,50,51]. Such morphological changes decrease tumor cell survival and tumor invasion. For instance, fasudil, a potent ROCK inhibitor, has been shown to inhibit colony formation and colony migration of tumor cells while inducing tumor cell death in a Rho-independent manner [48,52]. Similar results were demonstrated with treatment of Y27632 in melanoma cell lines including cutaneous and ocular melanoma, altering cell morphology such as limiting lamellipodia formation and decreasing cell invasiveness as well as cell survival in vitro [50]. This led to the prevention of tumor formation of the murine tumor model in vivo [48,50]. In pancreatic ductal adenocarcinoma (PDAC), AT13148 induced morphological changes from regular to irregular and reduced physical force generation. It also inhibited invasive behavior of tumor cells, thereby limiting tumor growth in vivo [49].

The Rho/ROCK signaling pathway has been said to involve several stages of cell mitosis and cytokinesis, resulting in a close association with the cell cycle [53]. Rho/ROCK is required for the accumulation of furrow cleavage during cytokinetic processes through phosphorylation of downstream proteins. Y27632 and fasudil prolonged ingression and cleavage furrow formation during cytokinesis [54,55]. Cells lacking in ROCK1 and ROCK2 showed lower number of division, an increase in both senescence-associated β-galactosidase and the percentage of G2/M cell cycle phase, suggesting that cell cycle was blocked and cellular senescence had been induced. Additionally, ROCK regulates various essential cell cycle related proteins including CyclinA, Cyclin-dependent kinases regulatory subunit 1 (CKS1), and Cyclin-dependent kinase 1 (CDK1), indicating that ROCK inhibition can lead to the suppression of tumor cell progression and tumorigenesis [37]. For example, liposomal fasudil has shown to prompt the antitumor effect by inducing G2/M phase cell cycle arrest in hepatocellular carcinoma (HCC) [56].

Along with cell cycle arrest, several ROCK inhibitors were shown to induce apoptosis in various tumor models [57]. Fasudil and Y27632 were found to induce apoptosis of the glioblastoma cell line in vitro. Fasudil also induced apoptosis of small cell lung cancer via reduction in c-myc and cyclin D expression, halting the cell cycle at the G2/M phase [58]. Furthermore, ROCK inhibitors not only induce apoptosis, but also autophagy. An analysis of Light Chain 3-II (LC3-II) protein levels indicating an increase in autophagosome formation, showed fasudil itself to be both an autophagy and apoptosis inducer. However, combination with another anti-cancer agent clioquinol showed synergistic effects on both the apoptosis pathway and autophagy, leading to a cytotoxic effect in U87 glioblastoma cells [59]. Similar effects were demonstrated on esophageal squamous cell carcinoma (ESCC) cells, where fasudil induced both ESCC cell apoptosis and autophagy. Interestingly, a combination of fasudil with autophagy inhibitor chloroquine enhanced the proapoptotic effect of fasudil [60]. This suggests a novel combinatory strategy to synergistically amplify the anti-cancer quality of fasudil, although further studies are required.

Rho/ROCK has also been proven to regulate DNA damage responses and repair mechanisms [61]. In melanoma, ROCK inhibitor treatment induced reactive oxygen species (ROS) generation by lowering the contractility of cancer cells, leading to DNA damage responses in vitro [62]. ROCK2 was also shown to be involved in centrosome complex formation with *BRCA2* in breast cancer, indicating the possibility that ROCK2 inhibition may induce DNA damage [63]. Correspondingly, ablation of ROCK2 sensitized gemcitabine-induced DNA damage via prevention of zinc finger E-box-binding homeobox 1 (ZEB1)-adipose tissue macrophage (ATM)/p-checkpoint kinase 1 (CHK-1) mediated DNA repair [64]. Increased expression of ROCK2 under the activation of Rho C was also confirmed to be involved with the DNA repair system in cervical cancer upon treatment with radiotherapy in vitro, rendering the tumor radioresistant, indicating that ROCK2 inhibition may affect the DNA damage repair response [65].

### 3.2. Inhibition of Tumor Metastasis, Invasion, and Migration

The involvement of Rho/ROCK in the metastatic ability of cancer cells is extensively studied in various ways. In particular, Rho/ROCK is largely involved in the cell migration process, controlling actomyosin contractility to exhibit an elongated cell morphology and a protrusive mesenchymal-like mode of migration [66,67]. Inhibition of actomyosin contractile force by ROCK inhibitors leads to changes in cell morphology and impairs cellular movement, associated with cell migration in prostatic cancer [51] and melanoma [68]. Additionally, high expressions of ROCK in HCC are associated with migration and invasiveness of cancer cells, leading to tumor metastasis [44,69,70]. On the other hand, knockdown of ROCK2 impairs cytoskeleton remodeling through activation of the ROCK- Myosin light-chain phosphatase 1 (MYPT1) and directional movement by inhibiting formation of filopodia and lamellipodia in HCC cells [44]. Another study has shown that ROCK-ezrin promotes HCC invasion. Ezrin phosphorylation at Thr567 promotes membrane ruffling, a characteristic feature of actively migrating cells to accelerate cell motility and invasiveness of HCC cells. Since ROCK is an upstream effector of ezrin, ROCK inhibition by either Y27632 or ROCK-targeted small interfering RNAs reduce Thr567 phosphorylation of ezrin, thereby limiting cancer cell metastasis [70].

Cancer cell metastasis is regulated by multiple proteins and genomic targets of ROCK downstream signaling pathways. Oncogenic c-Myc plays a pivotal role in the maintenance of tumorigenesis and metastasis by regulating microRNAs (miRNAs) such as miR-17-92 clusters [71,72,73]. ROCK1 stabilizes and phosphorylates c-Myc via direct interaction, controlling mRNA and miRNA clustering, and eliciting the metastatic and proliferative capabilities of prostate cancer [72] and breast cancer cells [71]. Treatment with Y27632 and siRNA molecules to inhibit ROCK mediated its downstream events and effectively prevented tumor growth and metastasis [71,72]. MLC, which is phosphorylated and activated by ROCK, controls the actin-myosin contraction and cytoskeletal reprogramming involved in cell morphology, motility, and metastatic process [74]. Hypoxia-inducible factors (HIF) directly bind to ROCK1 and phosphorylate MLC to mediate focal adhesion kinase (FAK) activation and support metastatic behavior of breast cancer cells under hypoxic conditions [75]. The growth factor endothelin-1 (ET-1)—endothelin type A or B receptors (ET_A_R and ET_B_R)—drive invadopodia formation to mediate actin-based invasive protrusion, resulting in cell invasion and metastasis. In epithelial ovarian cancer, the RhoC–ROCK–LIMK–Coflin pathway plays a key role in this process to mediate actin cytoskeleton reorganization and cell invasion due to the fact that the ET-1/ET_A_R axis promotes the interaction between β-arr1 and PDZ-RhoGEF, which is directly responsible for the activation of the RhoC–ROCK–LIMK–Coflin pathway [76].

Furthermore, ROCK is also associated with TME components to regulate the metastatic cascade. Tumor associated macrophages (TAMs) are one of the major contributors in the metastatic progression of breast cancer. Several studies have demonstrated that migration response of breast cancer cells due to TAMs was associated with Rho/ROCK signaling [77,78]. Subsequently, breast cancer cells that were ROCK-inhibited through treatment with Y-27632 or GSK429286A showed diminished migratory and invasive behaviors [78]. In addition, in some cancer types, glucocorticoids (GCs) were shown to regulate tumor cell progression and metastasis through the GC receptor [79,80]. Moreover, treatments with synthetic GCs such as dexamethasone or corticosterone promoted adhesion, migration, and metastasis of melanoma through the ROCK-phosphoinositide 3-kinase (PI3K)-AKT pathway, independent of Rho GTPases (RhoA, RhoB, and Rho C). ROCK inhibition by Y27632 abrogated such GC-mediated invasiveness of melanoma cells [80].

Progressive stages of EMT involve cytoskeletal changes and acquisition of migratory behavior and motility in cancer cells, pushing them to metastasize to other sites. ROCK plays a pivotal role in this EMT process, regulating microtubules, actomyosin contractility, and cytoskeletal changes, finally resulting in the loss of epithelial characteristics and the acquisition of mesenchymal ones [81,82]. A decrease in epithelial markers, an increase in tight junction formation to regulate cellular movement, and an upregulation of mesenchymal cellular phenotype markers facilitating cell motility such as N-cadherin, Snail, Slug, ZEB1, and Vimentin are common characteristics of the EMT process. Several studies have confirmed that inhibition of ROCK can prevent or reverse these changes in EMT markers [64,83,84]. One study demonstrated that the EMT phenotype in gemcitabine-resistant pancreatic cancer cells, commonly linked with chemo-resistant cells, were regulated upon ROCK2 inhibition [64]. Additionally, potent ROCK inhibition by treatment of GSK126 combined with diosgenein, a natural steroidal saponin, suppressed EMT process-associated molecules in gastric cancer cells [83]. Meanwhile, transforming growth factor (TGF)-β1 is one of the most pivotal signaling agents in promoting EMT by activation of the Smad complexes and targets gene transcriptional factors including Snail, ZEB, and Twist. Several studies determined the Rho/ROCK pathway to mediate TGF-β1-induced EMT, demonstrating how ROCK inhibition prevented TGF-β1-induced EMT marker changes and the corresponding morphological changes [84,85,86,87]. Furthermore, activation of the extracellular signal-regulated kinase (ERK)/p38 pathway as downstream of Rho/ROCK signaling was found to upregulate supervillin under hypoxic conditions, promoting EMT and metastasis in HCC [88]. In collagen-abundant pancreatic cancer, ROCK inhibitor AT13148 suppressed collagen invasion of the tumor by regulating cell motility and contractile force that requires actin polymerization, without affecting tumor cell proliferation [49].

### 3.3. ECM Remodeling

The TME consists of various cellular components that not only include cancer cells, but also vasculature, immune cells, and ECM. ECM supports tumor progression, migration, and invasion by ECM molecules, interacting with other TME components in autocrine and paracrine ways [89,90]. ROCK controls actomyosin contractility to drive ECM remodeling by deposition of ECM components [42,91]. Collagens are one of the most abundant and major proteins in the ECM, and in tumors, collagen deposition is increased in a ROCK-dependent way through contractile force generation, driving the deformation of collagen fibers and tissue stiffness, promoting tumor progression [92,93]. ROCK drives cell contractility to mediate fibrotic pathologies and fibrosis to stiffen the stroma, while cancer associated fibroblasts (CAFs) are the main source for the production of such ECM structural proteins [94]. In PDAC, ROCK1 was found to be overexpressed in tumor tissues and ROCK inhibition reverted the activated state of the pro-tumorigenic CAFs, accompanied by a reduction in collagen I expression. Moreover, a combination of fasudil with gemcitabine significantly decreased collagen deposition and enhanced anti-tumor efficacy [95]. Such impairment of collagen matrix by ROCK inhibitors effectively primed the TME of PDAC, sensitizing the tumor to chemotherapeutic agents such as gemcitabine or abraxane [96]. Another preclinical study also reported that treatment of fasudil primed tumor tissues by disrupting ECM integrity during ECM deposition and improving the chemotherapeutic efficacy of gemcitabine and abraxane in pancreatic cancers [96]. In breast cancer, ROCK downstream pathway protein kinase R-like ER kinase (PERK)-activating transcription factor 4 (ATF4) produces Creld2 protein, which educates fibroblasts in a paracrine way and mediates tumor progression through tumor–stroma crosstalk [97]. In multiple myeloma (MM), stromal cell–derived factor-1 (SDF1) and its receptor chemokine receptor 4 (CXCR4) induced MM cell adhesion to fibronectin, a ubiquitous ECM glycoprotein through the Rho/ROCK pathway. Inhibition of ROCK prevented such SDF1-induced adhesion of MM cells and their homing to the bone marrow [98,99].

### 3.4. Combinatory Effects with Other Chemotherapeutic Agents

Conventional chemotherapeutic drugs and treatment regimens in oncology show limited response rate and poor survival. In order to overcome certain chemotherapy resistance and improve the response rate, a combinatory strategy is necessary. Over the years, ROCK inhibitors have been suggested as a potential combinational partner to be used with existing treatments for several cancers. For example, fasudil was administered as a drug cocktail along with tranilast and temozolomide for neuronal reprogramming to improve the current most common treatment option for glioblastoma [100]. Furthermore, in pancreatic cancer, while gemcitabine is the first-line treatment agent, its efficacy is not ideal due to its limited response rate. Therefore, several studies have demonstrated that targeting ROCK may improve the sensitivity of gemcitabine in chemo-resistant pancreatic cancers [64,96,101]. In this manner, targeting ROCK is a possible approach to increasing drug sensitivity of existing chemotherapeutic agents.

There are further examples of research demonstrating the combinatory potential of ROCK. Genetic screening has found ROCK to be a promising combinative agent with EGFR inhibitors to produce synergistic antitumor responses against a triple negative breast cancer (TNBC) model. This dual inhibition of EGFR and ROCK mediated an increase in the G2 phase of the cell cycle and a loss of Cyclin A and Cdk2 as well as p27Kip1. Combined EGFR and ROCK inhibition impaired TNBC cell growth and induced synergistic pharmacological effects through cell cycle arrest by decreasing Cyclin–Cdk complex-mediated phosphorylation of pRb [102]. Another example of ROCK inhibitor combination was found through a synthetic lethal drug screening, and co-treatment of polo-like kinase 1 (PLK1) and ROCK inhibitors showed synergistic therapeutic efficacy in *KRAS*-mutant cancers. In particular, this combination specifically upregulated the genomic and protein levels of p21^WAF1/CIP1^ in *KRAS*-mutant cells, leading to a G2/M cell cycle arrest and impairing cell survival through apoptosis [103]. In addition, addition of ROCK inhibitors was suggested as a strategy to overcome the resistance of mitogen-activated protein kinase (MEK) inhibitors against *NRAS* mutant melanoma. This combination of MEK inhibitors and ROCK inhibitors was shown to cooperatively lower the mitogen-activated protein kinase (MAPK)/ERK downstream signaling pathway and upregulate cyclin-dependent kinase inhibitors including p16^INK4A^, p21^CIP1^, and p27^KIP1^ as well as pro-apoptotic signaling in protein levels to induce cellular cytostatic and apoptotic response [104]. Another study interestingly showed that neuronal damage caused by cisplatin through cytokine releases induced by cisplatin can be prevented upon Y27632 treatment [105]. In conclusion, there has been multiple studies validating the use of ROCK as a combinatory tool in increasing drug sensitivity, antitumor responses, or even preventing side effects.

## 4. ROCK Regulates Immune Cells

### 4.1. Dendritic Cells and Macrophages

Dendritic cells (DCs) and macrophages are representative antigen presenting cells, specializing in the detection, engulfment, and presentation of antigens to mediate adaptive immune responses. During the engulfment process in phagocytes, Rho activity is downregulated and Rho/ROCK signaling acts as a negative regulator for complement receptor mediated phagocytosis [106]. Correspondingly, Rho/ROCK inhibition in phagocytes mediates apoptotic cell engulfment [107,108]. Moreover, motor protein myosin IXB (Myo9b), an upstream negative regulator of the Rho/ROCK signaling pathway, affects the chemotaxis and migratory behavior of macrophages [109] and DCs [110], leading to their loss of function concerning antigen presentation to T-cells, thereby failing to effectively induce adaptive immune response [110]. Rho/ROCK signaling also induced morphological changes of DCs to extend dendrites, which was associated with actin polymerization. Inhibition of Rho/ROCK augmented the production of interleukin-12 (IL-12) of DCs and when these DCs were cultured with T cells, there was an increase in interferon-γ (IFN-γ)-producing CD4^+^ T cells. Interestingly, there was a significant decrease in IL-2-producing T-cells [111]. In addition, Rho/ROCK is known to be essential for Toll-like receptor 2 (TLR2)-mediated IL-23 inflammatory immune response in rheumatoid arthritis macrophages [112]. M2-like macrophages are suggested to be linked to macular degeneration, since they are found to be accumulated in age-related macular degeneration (AMD), but not in normal eyes. Selective ROCK2 inhibition has been shown to reduce M2-like macrophage subtypes and choroidal neovascularization. ROCK2 inhibition also upregulated M1 markers with no effect on macrophage recruitment, drawing attention to the role of ROCK2 in macrophage plasticity [113]. Another study demonstrated that vascular endothelial growth factor (VEGF)/chemokine ligand 18 (CCL18) from IL-4/IL-13 induced M2a type macrophage promoted the invasion of breast cancer cells via the Rho/ROCK signaling pathway, and this could be attenuated by a ROCK inhibitor [78].

### 4.2. T-Cells

Rho-kinase signaling has proven to be important in the induction of T-cell immune dysfunction in abdominal sepsis by regulating sepsis-induced systemic inflammation. In septic animals, Rho-kinase inhibitor pretreatment not only helped T-cell functionality by decreasing apoptosis and increasing CD4^+^ T-cell proliferation, but also abrogated the systemic bacteremia. This indicates that Rho-kinase may be involved in improving host defenses mediated by T-cells in the case of abdominal sepsis. When cecal ligation and puncture (CLP) induced apoptosis of splenic CD4^+^ T-cells and the increase in splenic regulatory T-cells (T_reg_), Rho-kinase inhibition effectively abolished such effects [114]. Furthermore, regulation of T-cell cytoskeleton is essential for the formation of immune synapses. Inhibition of ROCK in naïve T-cells led to their activation by remodeling actin through the ROCK/LIMK/Cofilin signaling pathway [115]. In addition, ROCK expression in T-cells has been found to be mostly located at the trailing edges of T-cells when they migrate, regulating the detachment of T-cells [116].

Inhibition of ROCK was shown to alleviate the pathogenesis of immunopathogenic diseases where T helper 17 cells (T_h_17) plays a pivotal role [117]. Recent studies have illustrated the role of ROCK2, but not ROCK1, in the differentiation of T_h_17 cells, which mediates inflammatory responses in autoimmune disorders through the janus kinase (JAK)/signal transducer and activator of transcription (STAT) pathway [118]. ROCK2 has also been established as a regulator of IL-21 and IL-17 secretion of human T-cells through STAT3, interferon regulatory factor 4 (IRF4), and RAR-related orphan receptor γt (RORγt) regulation [119,120]. Another study demonstrated that an oral formulation ROCK2-selective inhibitor, KD025, increased T_reg_ function by increasing STAT5 phosphorylation, concluding that ROCK2 is crucial in modulating human immune homeostasis, and that selective ROCK2 inhibition shifts the T_h_17/T_reg_ balance toward the T_reg_ phenotype [121].

### 4.3. Natural Killer Cells (NK Cells)

Rho-kinase regulates actin dynamics, associated with membrane kinetics to alter cellular morphology by the formation of membrane protrusion including lamellipodia, ruffling, and blebs [122]. Use of a ROCK inhibitor converted the killing mode of NK cells from necrotic mode to death ligand-mediated apoptosis in necrosis sensitive MCF7 cells by reducing membrane blebs with lamellipodia extension. On the other hand, in SMMC-7721 cells, which have a moderate membrane dynamics with intrinsic apoptosis sensitivity, the killing mode of NK cells was not affected [123]. The RhoA/ROCK/LIM-kinase pathway in NK cells involves actin cytoskeletal reorganization and lipid raft polarization to form immunological synapse of NK cell-target cell, and thereby induce the cytotoxic activation of NK cells. Incubation of NK cells with a ROCK inhibitor was found to lower their cytotoxic effect in a concentration-dependent manner [124]. Furthermore, the ROCK-AKT signaling pathway is also associated with NK cell activity. NK cells treated with ROCK inhibitors showed PI3K-dependent AKT activation and have enhanced cytotoxic activity against different cancer cell lines [125].

### 4.4. Other Immune Cells

Lymphotoxin beta receptor (LTβR) ligands on DCs are crucial in lymph node immune responses. This signaling between DCs and reticular cells mediates cell survival by modulating podoplanin (PDPN). PDPN regulates integrin-mediated cell adhesion, maintaining reticular cell survival. In vitro treatment with a Rho-kinase inhibitor blocked PDPN downstream effects and disrupted cell survival, which shows that PDPN regulates reticular cell survival via the Rho–ROCK–ERM pathway that is usually related to cell contractility [126]. Therefore, ROCK plays an important part in maintaining the DC-stromal axis shifting toward the continuation of immune response and lymphocyte survival.

The relationship between ROCK and monocytes are also well-known. Monocyte chemoattractant protein-1 (MCP-1) is a key chemokine in the recruitment of monocytes. While TNF-α has been shown to induce MCP-1 expression in mesangial cells, treatment of Y-27632 inhibits this TNF-α-dependent monocyte migration [127]. Another recent study demonstrated the effect of ROCK2 in the recruitment of monocytes. Silencing of ROCK2 effectively inhibited the migration and adhesion of monocytes to endothelial cells by attenuating nuclear factor κB (NFκB)-dependent induction of chemokines and cell adhesion molecules [128]. Both of these studies establish the key role ROCK plays in the recruitment of monocytes.

## 5. ROCK and Cancer Immunotherapy

**Figure 3:**
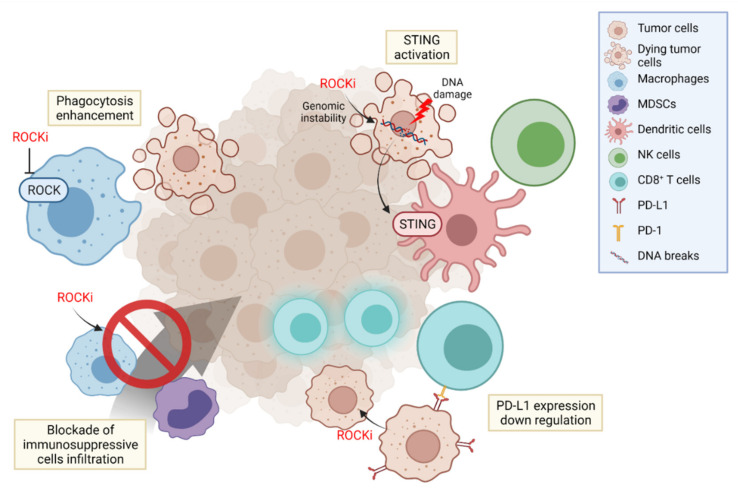
Immunotherapeutic strategies using ROCK inhibitors. ROCK inhibition in antigen presenting cells upregulates their phagocytic function to eliminate cancer cells and mediates the adaptive immune response. In addition, blockade of ROCK in cancer cells can maintain enough genomic instability to activate the cGAS/STING pathway and evoke sequential immune cell responses. ROCK inhibition can also hamper the PD-L1 expression in cancer cells and persist T-cell activation. YAP nuclear localization is controlled by Rho/ROCK signaling to recruit immunosuppressive cells in the TME. Furthermore, ROCK inhibitors can alter the TME by restricting the infiltration of MDSCs or M2 type macrophages.

In the TME, various immune cells are constantly involved in tumor progression and suppression. Cancer immunotherapy targets these immune cells in the cancer immunity cycle, blocking each step of cancer development [129]. Thus, it is important to adopt a proper immunotherapeutic strategy based on the timing, sequence, combination, and delivery of immunotherapeutic agents [130]. Here, we provide evidence to validate our opinion of ROCK as an effective immunotherapeutic target to treat cancer in multiple stages (Figure 3).

### 5.1. Regulation of Phagocytosis

Phagocytosis is an initial and fundamental process to detect and eliminate tumor antigens, provoking an anti-tumor immune response. This process bridges the innate and adaptive immunity by priming T-cells to recognize tumor cells and have a killing effect. The elimination of apoptotic cells through phagocytosis was proven to be negatively regulated by RhoA/ROCK signaling, [131] and blocking the RhoA/ROCK pathway enhanced the phagocytic activities of macrophages [108]. Accordingly, use of ROCK inhibitor Y27632 increased the phagocytic activity of antigen presenting cells, inducing their tumor cell uptake and antigen presenting capability, proving that ROCK inhibition can effectively potentiate the innate immune response [132]. Furthermore, in addition to this capacity of ROCK inhibitors of enhancing antigen presentation, a combination with immunogenic cell death (ICD) inducers such as doxorubicin or photodynamic therapy showed a synergistic effect in suppressing tumor growth. This combination of ‘Eat me’ signal increase by ICD inducers and the augmented phagocytosis by ROCK inhibitors collectively boosted antitumor immunity, effectively recruiting CD8^+^ T-cells, sensitizing the immune-excluded or immune desert tumors to improve their response toward immune checkpoint blockade therapies [132,133].

### 5.2. Activating Innate Immune System

Genomic instability is one of the key indicators in evaluating immunotherapy response [134]. Interference in the DNA repair system causes DNA damage, leading to the formation of single-stranded or double-stranded DNA (dsDNA) breakage. In particular, dsDNA activates the cyclic GAMP-AMP synthase (cGAS)/stimulator of interferon genes (STING) pathway, promoting type I interferon secretion and activating the innate immune system. Numerous agents such as poly (ADP-ribose) polymerase (PARP) inhibitors have been developed in an attempt to induce DNA breakage and activate the cGAS/STING pathway [135]. Furthermore, activation of the cGAS/STING pathway using a STING agonist potentiated NK cell immunity through the secretion of IL-15 from DCs activated by IFN-γ from the myeloid cell population [136]. In this fashion, there have been numerous efforts to induce the cGAS/STING pathway in order to trigger an immune response to utilize in cancer immunotherapy.

Since it is well-known that ROCK can cause cell cycle arrest and maintain DNA damage, there seems to be a possible potential in using ROCK inhibitors to interrupt the DNA repair system. An upstream signaling molecule of ROCK, Rho A, is closely related to DNA damage response, suggesting the conceivable role of ROCK in the DNA repair system [137]. However, there is still a lack of evidence and further studies are necessary in order to prove this hypothesis.

In addition to its ability to retain DNA damage, the role of ROCK in tumor senescence is also interesting. As mentioned before, the inhibition of ROCK has been proven numerous times to induce cellular senescence of tumors, and this effect on the immune system is worth considering. For example, the induction of cellular senescence of *KRAS*^mut^ tumors has been effective in the suppression and elimination of tumor cells due to an increase in senescence-induced secretory phenotype via NK cells [138]. In a similar way, ROCK inhibition-induced cellular senescence of tumors may have a potential in invigorating NK cells to activate a senescence-mediated antitumor immunity.

### 5.3. PD-L1 Depletion

PD-L1 is an immunotherapeutic target that suppresses T cell activation. PD-L1 expression on tumor cells and tumor infiltrating immune cells are crucial for tumors in dampening T-cell responses and thereby escaping the immune system. Therefore, understanding the regulatory mechanisms of PD-L1 expression and targeting them may offer therapeutic innovations in cancer immunotherapy. In breast cancer, PD-L1 was shown to be stabilized by moesin phosphorylation induced by ROCK. Such phosphorylated moesin competes with E3 ubiquitin ligase to bind with PD-L1, inhibiting its degradation. Naturally, blocking ROCK in the mechanism has the potential to interfere with PD-L1 stability and upregulate the immune response in breast cancer [139].

It has also been demonstrated that TGF-β induces PD-L1 expression to contribute to the immune evasion of cancer cells. In this axis, Myocardin-related transcription factor-A (MRTF-A) plays an immune suppressive function. Rho/ROCK signaling is involved in the noncanonical pathway to suppress TGF-β induced PD-L1 expression, contributing the translocation of MRTF-A to the nucleus [140].

These results suggest another mechanism of ROCK inhibition as a cancer immunotherapeutic strategy that can potentiate T-cell activity.

### 5.4. Overcoming Resistance to Immunotherapy

The RAS signaling pathway is known to be highly active in melanoma, and chemotherapeutic or immunotherapeutic agents such as BRAF inhibitors, MAPK inhibitors, or MEK inhibitors targeting this pathway have been actively studied. However, they have been shown to fail due to chemoresistance mediated by myosin II activation [141]. This leads to the rapid migration of melanoma and the induction of immunosuppressive secretome such as IL-1a, modulating the TME by reducing immunosuppressive differentiation of myeloid cells such as M2-like macrophages [142]. Such resistance can be overcome by ROCK inhibitors that reduce myosin II activity, leading to DNA damage by generating ROS in immunotherapy-resistant melanoma and decrease in PD-L1 and T_reg_ infiltration [143]. In addition, melanoma cells pre-treated with ROCK inhibitors have shown suppressed tumor growth through the increase in the Fas ligand (FasL) and the corresponding infiltration of CD8^+^ T lymphocytes [144]. Overall, these studies offer another potential of ROCK inhibitors in melanoma immunotherapy.

### 5.5. YAP Inhibition

The hippo signaling pathway plays a critical role in promoting the migration, invasion and malignancy of cancer cells, therefore bearing a unique tumorigenic capacity. Most of these activities are mediated by the transcriptional effector yes associated protein (YAP) and its paralog transcriptional coactivator with PDZ-binding motif (TAZ) through their interaction with transcriptional factors [145]. ROCK is involved in maintaining the nuclear localization of YAP, thereby upregulating YAP activity [146]. For example, Rho-signaling was found to be necessary for human embryonic stem cell survival, since ROCK sustains the nuclear function of YAP/TAZ [147]. On a similar note, deprivation of ROCK effectively reduced the expression of YAP in a dose-dependent and time-dependent manner, inhibiting the growth and metastatic ability of osteosarcoma cells [148].

The hippo signaling pathway not only plays a part in tumor cells, but also has an immunomodulatory effect and orchestrates a number of immune cells in the TME. Furthermore, YAP also plays a critical part in suppressing anti-tumor immunity, especially through T-cells. For example, suppression of the hippo pathway was shown to subdue CD8^+^ T-cell differentiation [149]. In addition, YAP was found to be expressed by activated CD8^+^ T-cells found in the TME, acting as an immunoinhibitor on these cytotoxic cells [150]. YAP was also found to be upregulated in T_reg_ cells, playing a crucial role in directing T_reg_ function through the amplification of TGF-β [151] and promoting T_reg_ differentiation through the upregulation of *TGFBR2* expression [152]. Another recent study identified YAP as a broad suppressor of CD4^+^ and CD8^+^ T-cells, and a critical regulator of T-cell tumor infiltration and patient survival [153]. YAP is also closely related to other immune cells. For example, in prostate adenocarcinoma models, MDSCs were recruited to the TME to promote cancer progression in a YAP-dependent manner [154]. Not only so, in *KRAS*:*p53*-mutant PDAC, YAP promoted the differentiation of MDSCs, making them capable of impairing T-cell activation [155]. In the case of TAMs, YAP was found to increase M2 TAM polarization in CRC, and use of a YAP inhibitor suppressed this tumor-initiating polarization and subsequent tumorigenesis [156]. Due to its relationship with various immune cells, high YAP expression in tumors is already considered as an indicator of poor immunologic prognosis, for example, in CRC, due to the effect of YAP on MDSCs and M2 TAM polarization [156,157].

YAP seems to be an important target in improving T-cell response in cancer, furthermore in improving cancer immunotherapy response. Naturally, therapeutic interventions to prevent such YAP-dependent TME suppressions are currently under research. For example, YAP was found to regulate tumoral *PD-L1* expression at the transcriptional level in non-small cell lung cancer and BRAFi-resistant melanoma [158,159]. This role of YAP in promoting PD-L1 in cancer cells provides an avenue of possibility to enhance PD-L1 immune checkpoint blockade therapy. Indeed, YAP inhibition directly hindered the expression of PD-L1, providing an effective strategy to overcome gefitinib-resistant lung adenocarcimoma [160]. The use of verteporfin, a YAP inhibitor, with PD-1 antibodies resulted in a synergistic reduction in tumor growth, validating the opinion that YAP suppression can boost the efficacy of immunotherapies. In addition, YAP inhibition alone and in combination with 5-fluorouracil (5-FU) suppressed TAM infiltration, polarization, and TAM-associated resistance toward 5-FU treatment, resulting in an increase in therapeutic response in patients [156].

Taking into account the role ROCK plays in the regulation of YAP expression, we suggest ROCK as an indirect regulator of YAP-mediated immune cell interference. Furthermore, modulation of ROCK may lead to an increase in immune response, and therefore could become a useful tool in cancer immunotherapy.

## 6. Conclusions and Perspectives

ROCK contributes to a variety of cytoskeletal-associated functions in cells by regulating the actin cytoskeleton. In cancer, ROCK plays multiple roles in cell survival, invasion, metastasis, EMT remodeling, and drug resistance. Therefore, targeting ROCK has become a promising approach in controlling tumor progression, leading to the launch of a ROCK inhibitor to a phase I clinical trial with the indication for cancer. However, most studies on ROCK in the field of cancer therapy have been focused on the direct effectiveness of ROCK on cancer cells, rather than its surrounding components. Thus, the specific role of ROCK in TME components still needs to be elucidated through further studies.

With the emergence of cancer immunotherapy, identifying novel targets to control the TME in order to evoke anti-cancer immune responses has become imperative. In that sense, as a modulator of multiple functions in various cells, ROCK is a promising drug target for cancer immunology. As described, ROCK plays multiple roles in regulating various immune cells in many diseases. Depending on the immunopathology of the diseases, ROCK may play contradictory roles. For example, in an immune-inflamed environment, ROCK inhibitors could induce immunosuppressive cells such as T_reg_. On the other hand, in an immunosuppressive environment such as the TME, ROCK inhibitors could hinder the recruitment of immunosuppressive cells such as MDSCs or TAMs. Furthermore, discovery of novel small-molecule inhibitors of ROCK have provided numerous opportunities to study ROCK as a cancer treatment modality. However, several key requirements need to be met with these ROCK inhibitors in order to establish their position as a method of cancer treatment including targetability, selectivity, and activity. For example, targeting one isoform of ROCK or both may offer different, even conflicting functional roles in cellular activity.

Therefore, a deep insight into the mechanistic properties of ROCK is required. Given all the above, ROCK is an attractive target for cancer immunotherapy. Further research supporting the rationale to utilize ROCK inhibitors in appropriate circumstances and in combination with other therapeutic strategies may provide greater clinical benefits in immunotherapy resistant patients.

## Figures and Tables

**Table 1:** Ongoing clinical trials targeting Rho-kinase in various indications ^1^.

Drug	NCT Number	Indication	Phase	Status	Note
AR-12286 (Verosudil)	NCT01330979	Open Angle Glaucoma, Ocular Hypertension (OH)	2	Completed	
	NCT01699464		2	Completed	
	NCT01936389	Exfoliation Syndrome, OH, Open Angle Glaucoma	2	Unknown	
	NCT02174991	Glaucoma	2	Unknown	
	NCT01060579		2	Completed	
	NCT02173223	Advanced Glaucoma	2	Unknown	
	NCT02152774	Chronic Angle-closure Glaucoma	2	Unknown	
	NCT00902200	Elevated Intraocular Pressure	2	Completed	
	NCT01302249	Glaucoma, OH	2	Completed	Latanoprost
	NCT01474135		2	Completed	Travoprost
Fasudil		Cerebral Vasospasm, Cerebral Ischemic Symptoms	Approved in Japan (June, 1995)		
	NCT01935518	Amyotrophic Lateral Sclerosis	2	Unknown	
	NCT03792490		2	Recruiting	
	NCT00670202	Carotid Stenosis	2	Terminated	
	NCT04734379	Progressive Supranuclear Palsy, Corticobasal Syndrome	2	Recruiting	
	NCT04191954	Retinopathy of Prematurity	2/3	Recruiting	
	NCT04793659	Dementia	2	Active, not recruiting	
	NCT00498615	Raynaud’s Disease, Scleroderma	3	Completed	
	NCT01823081	Diabetic Macular Edema	3	Completed	
	NCT03753269	ST Segment Elevation, Myocardial Infarction	4	Not yet recruiting	
	NCT00120718	Atherosclerosis, Hypercholesterolemia	2	Completed	
	NCT03404843	Cardiovascular Diseases	2	Completed	
	NCT03391219	Retinal Vein Occlusion	2/3	Unknown	bevacizumab
	NCT04734379	Progressive Supranuclear Palsy, Sorticobasal Syndrome	2	Recruiting	
Ripasudil		Glaucoma, OH	Approved in Japan (September, 2014)		
	NCT03575130	Fuchs’ Endothelial Dystrophy, Fuchs Dystrophy, Corneal Endothelial Dystrophy, Corneal Endothelial Cell Loss, Cornea Guttata	2	Unknown	
	NCT03813056	Fuchs’ Endothelial Dystrophy	2	Recruiting	
	NCT03249337		4	Recruiting	
	NCT04621136	Retinopathy of Prematurity	1/2	Recruiting	
Netarsudil		Open-Angle glaucoma, OH	Approved (2019)		Latanoprost
	NCT04057053	Fuchs’ Endothelial Dystrophy, Cataract	Early 1	Completed	
	NCT04752020	Fuchs’ Endothelial Dystrophy	Early 1	Recruiting	
	NCT03248037	Fuchs’ Endothelial Dystrophy, Bullous Keratopathy	3	Completed	
	NCT04620135	Primary Open Angle Glaucoma, OH	3	Completed	
	NCT03233308		2	Completed	
	NCT04064918	Open-Angle Glaucoma, OH	Not applicable	Not yet recruiting	
	NCT02558374		3	Completed	
	NCT02874846		2	Completed	
	NCT02246764	OH, Glaucoma	3	Completed	
	NCT04498169	Corneal Edema	2	Completed	
KD025 (Belumosudil)		Chronic Graft-Versus-Host Disease (cGVHD)	Approved (2021)		
	NCT03640481	cGVHD	2	Recruiting	
	NCT02841995	GVHD	2	Active, not recruiting	
	NCT02317627	Psoriasis Vulgaris	2	Completed	
	NCT02106195		2	Completed	
	NCT03907540	Autoimmune Diseases, Fibrosis	1	Completed	
	NCT02688647	Idiopathic Pulmonary Fibrosis	2	Completed	
	NCT03919799	Systemic Sclerosis, Diffuse Cutaneous Systemic Sclerosis	2	Recruiting	
	NCT04166942	Hepatic Impairment	1	Recruiting	
AT13148 ^2^	NCT01585701	Advanced Solid Tumor	1	Completed	

^1^ Data in this table are based on the advanced search of the Rho-kinase inhibitor except AT13148 on the NIH ClinicalTrials.gov site. (https://clinicaltrials.gov/, accessed on 19 October 2021) ^2^ AT13148 is applied to a clinical trial as a novel cyclic adenosine monophosphate (cAMP)-dependent, cyclic guanosine monophosphate (cGMP)-dependent, and protein kinase C (AGC) inhibitor.
